# Development and validation of the embodied cognition scale for Chinese university students

**DOI:** 10.3389/fpsyg.2025.1682631

**Published:** 2025-11-10

**Authors:** Ziyu Zheng, Yu Wang, Jian Zhao

**Affiliations:** 1 Music and Dance Academy of Heze University, Heze, China; 2 Music and Dance Academy of Heze University, Taishan University, Tai'an, China

**Keywords:** embodied cognition, psychological resilience, environment, activity patterns, university students, mediation model, structural equation modeling, well-being

## Abstract

**Introduction:**

Embodied cognition theory emphasizes that cognition is grounded in bodily movement, perception, and interaction with the environment rather than being purely symbolic or abstract. However, most existing embodied cognition measures are Western-based and lack cultural adaptability. This study aimed to develop and validate the Embodied Cognition Scale (ECS) tailored for Chinese university students, integrating both psychological and cultural dimensions of body–mind unity.

**Methods:**

A mixed-method design was employed across three sequential phases: (1) qualitative exploration with 27 students and 8 teachers to identify embodied cognition patterns; (2) exploratory factor analysis (EFA) and item refinement with 465 participants; and (3) national validation with 918 participants across multiple regions, using confirmatory factor analysis (CFA) and criterion validity testing with the Body Consciousness Scale.

**Results:**

EFA revealed a five-factor structure—bodily perception, social embodiment, embodied imitation, emotional embodiment, and cognitive reconstruction—explaining 61.15% of total variance. The model demonstrated good psychometric properties (Cronbach’s *α* = 0.954; CFI = 0.928; RMSEA = 0.062) and strong external validity (*r* = 0.81, *p* < 0.001). Test–retest reliability (*r* = 0.94) confirmed stability over time.

**Discussion:**

The ECS offers a culturally grounded, psychometrically robust tool for assessing embodied processes in higher education. By integrating Confucian concepts of body–mind unity and national education modernization goals, it provides both a theoretical and practical framework for embodied learning and mental health promotion among Chinese university students.

## 1 Introduction

### 1.1 Research background

The theory of Embodied Cognition proposes that cognition is not merely an abstract, symbolic process within the brain but is grounded in bodily movement, sensory experience, and continuous interaction with the environment (Barsalou, 2020; Ye, 2011). This theoretical stance redefines cognition as a mind–body–environment system, in which bodily sensations shape perceptual, emotional, and social understanding. It aligns with the long-standing Confucian principle of “unity of body and mind,” thus providing a unique cultural foundation for research in the Chinese educational context.

Empirical studies have consistently shown that physical movement can facilitate flexible and creative thinking. For instance, enactive tasks such as “breaking virtual walls” enhance divergent thinking by reducing over-control in the dorsolateral prefrontal cortex (Wang et al., 2019), confirming that motor actions restructure cognitive strategies. Meanwhile, open body postures can induce positive affect (Hao et al., 2017), and embodied interventions such as dance therapy promote post-traumatic growth through non-verbal bodily expression (Cheng, 2022). These findings support the notion that bodily actions regulate cognition and emotion bidirectionally.

However, the current tools for measuring embodied cognition remain limited and culturally biased. Most existing scales originate from Western cognitive paradigms (Shapiro, 2011; Glenberg, 2010), emphasizing visual–motor tasks while underrepresenting emotional embodiment and cultural variation in body awareness. As scholars note, cultural factors fundamentally shape the ways individuals perceive, express, and conceptualize bodily experience (Gallese, 2023). Therefore, Western scales may inadequately capture the embodied cognitive patterns of Chinese learners, whose bodily practices are deeply influenced by collectivist values, aesthetic education, and moral cultivation traditions.

Furthermore, traditional psychometric approaches often neglect the situated and generative nature of cognition (Thelen and Smith, 1994). Standardized “disembodied” assessments fail to reflect the adaptive and context-sensitive features of bodily engagement in real learning environments. To address this gap, developing a culturally adapted embodied cognition scale is essential for accurately assessing how Chinese students integrate body, emotion, and environment in daily academic and social life.

Importantly, this research responds to the national agenda articulated in China Education Modernization 2035 (State Council of the People’s Republic of China, 2019), which emphasizes the transformation of evaluation paradigms from knowledge-based assessment to integrated mind–body development. By creating a standardized scale grounded in the local embodied culture, this study aims to advance both theoretical understanding and practical applications in education and mental health, offering a quantitative basis for embodied interventions, active learning, and art-based psychosocial programs in Chinese universities.

### 1.2 Theoretical basis and dimension construction

The theory of embodied cognition posits that cognitive processes are fundamentally grounded in the physical characteristics and sensorimotor capacities of the body (Barsalou, 2008, 2020; Shapiro, 2019). The human body not only constrains but also enables cognition, determining how individuals perceive, act, and conceptualize the world. For instance, the bipedal structure of humans shapes their perception of vertical space, while the sonar system of bats constructs an entirely different perceptual modality (Ye, 2011). Cognitive representations are thus inherently body-dependent and emerge from continuous bodily engagement with the environment.

#### 1.2.1 Core sub-theories and their convergences

Contemporary scholarship distinguishes several complementary strands within embodied cognition: Embodied cognition emphasizes that thought and conceptual understanding arise from bodily experiences and sensorimotor patterns (Lakoff and Johnson, 1999; Niedenthal, 2007). Enactive cognition argues that cognition is not representation-driven but enacted through dynamic interaction between an organism and its environment (Varela et al., 1991; Di Paolo et al., 2017). Extended cognition expands this interactionist view by suggesting that external artifacts and environments function as cognitive components (Clark and Chalmers, 1998; Rowlands, 2009). Situated cognition highlights that cognitive performance is context-dependent, emerging within specific cultural, social, and environmental settings (Wilson and Golonka, 2013; Glenberg, 2010).

These approaches share a common rejection of “disembodied” mentalism but differ in emphasis: embodied focuses on bodily grounding, enactive on self–environment co-constitution, extended on cognitive off-loading to the environment, and situated on contextual learning and practice. Together, they provide a multidimensional foundation for scale construction, enabling assessment from physiological, environmental, emotional, and cognitive levels.

#### 1.2.2 The body as the material basis of cognition

The body provides the biological substrate for cognition. Empirical studies indicate that bodily posture, movement, and interoception influence memory encoding, attention, and reasoning (Craig, 2009; Damasio, 1999). For example, sensorimotor participation enhances conceptual learning (Barsalou, 2020), while muscular tension alters affective judgment (Strack et al., 1988). Ye (2011) further noted that bodily perception restricts the scope of cognitive representation, and Wang (2022) demonstrated that students construct self-understanding through somatic awareness in aesthetic education. These findings collectively substantiate the “body-as-cognition” assumption.

#### 1.2.3 The environment as a constituent of cognition

Cognition is distributed across body and environment. The extended mind hypothesis (Clark and Chalmers, 1998) and Rowlands’ (2009) ecological perspective emphasize that external tools and spaces form part of the cognitive system. Zhuang (2024) proposed the principle of “integration of body and context” in dance pedagogy, highlighting how environmental affordances modulate understanding. Similarly, research in cognitive neuroscience reveals that contextual cues dynamically reorganize neural activation during action perception (Gallese, 2023). In the Chinese context, the Confucian notion of “heaven–earth–human unity” resonates with this relational perspective, suggesting that embodied cognition is inherently ecological and cultural.

#### 1.2.4 Emotion and embodiment

Emotion is both embodied and regulatory. The emotion–body bidirectional mapping hypothesis (Niedenthal, 2007) demonstrates that emotional understanding involves facial-muscle activation and bodily resonance. Mind–body feedback loops play crucial roles in emotional regulation (Kabat-Zinn, 2003). Recent cross-disciplinary evidence from dance movement therapy and affective neuroscience confirms that expressive movement reorganizes emotional processing pathways (Bräuninger, 2014; Cheng, 2022). In Chinese art-education settings, Li and Xiong (2021) observed that backward movements evoke alertness, illustrating how bodily motion modulates psychological states. Emotional embodiment thus bridges sensorimotor and affective systems, providing a critical mechanism for mental health education (Ye and Su, 2024).

#### 1.2.5 Cognitive reconstruction through bodily action

Physical activity facilitates cognitive flexibility and creative insight. From a neurocognitive standpoint, action execution can inhibit excessive top-down control from the prefrontal cortex, thereby enhancing divergent thinking (Wang et al., 2019). Educational research identifies movement-based improvisation as a means of restructuring conceptual knowledge (Li and Xiong, 2021; Zhang, 2024). This aligns with Glenberg’s (2010) assertion that bodily engagement provides the generative mechanism for abstract reasoning. Accordingly, cognitive reconstruction in the current model captures how bodily movement mediates learning, meaning-making, and adaptive thought.

#### 1.2.6 Integrative and cultural model

Integrating these strands, this study conceptualizes embodied cognition as a five-dimensional system encompassing bodily perception, social embodiment, embodied imitation, emotional embodiment, and cognitive reconstruction. The model not only synthesizes Western theoretical advances but also embeds Chinese philosophical and pedagogical values—particularly the unity of knowing and doing emphasized in China Education Modernization 2035 (State Council of the People’s Republic of China, 2019). This perspective reframes embodiment as both a universal biological mechanism and a context-sensitive cultural practice, underscoring the need for a localized assessment tool to capture embodied learning in Chinese higher education.

#### 1.2.7 Addressing theoretical tensions and critiques

While the embodied cognition paradigm has significantly advanced psychological theory, it faces several conceptual and methodological critiques. Scholars argue that EC risks over-physicalizing cognition, neglecting abstract symbolic reasoning (Mahon and Caramazza, 2008), and that empirical studies sometimes lack consistent operational definitions (Wilson and Golonka, 2013). Moreover, quantifying bodily experiences across cultures poses measurement challenges (Shapiro, 2019). By incorporating cultural-psychometric principles and mixed methods, the present study seeks to mitigate these limitations and contribute to a more contextualized, measurable, and integrative framework for embodied cognition.

### 1.3 Embodied cognition and mental health

The theory of Embodied Cognition profoundly reveals the essential interdependence of the body and mind in sustaining psychological health. Cognition, in this view, is not an abstract symbolic computation isolated within the brain but a dynamic system that emerges through continuous bodily interaction with the physical, social, and cultural environment (Barsalou, 2020; Ye and Su, 2024). This paradigm challenges the traditional Cartesian dualism and asserts that bodily postures, gestures, and sensory experiences actively shape emotional states and mental resilience (Shapiro, 2019).

Recent studies in clinical and health psychology have demonstrated that bodily engagement serves as an effective medium for emotion regulation and stress recovery. For example, posture adjustment can enhance self-efficacy and reduce anxiety through autonomic feedback (Peper et al., 2021); and mindfulness-based body scanning improves interoceptive awareness and resilience by fostering attention to bodily sensations (Kabat-Zinn, 2003; Mehling et al., 2022). In dance movement therapy, the process of expressing implicit memories through structured bodily movement leads to post-traumatic growth and improved affective regulation (Bräuninger, 2014; Cheng, 2022). Similarly, occupational therapy research confirms that rhythmic motor routines and embodied task performance can restore self-agency in patients recovering from depression or trauma (Koch et al., 2019).

In the context of higher education, the Embodied Cognition framework provides a novel perspective for promoting university students’ mental well-being. Rapid social transformation, academic competition, and digital lifestyles have increased students’ cognitive overload and physical inactivity, leading to emotional exhaustion and reduced body awareness (Ding, 2023; Ye and Su, 2024). Embodied educational practices—such as somatic learning, movement-based reflection, and expressive arts—help re-integrate sensory, cognitive, and affective processes, thereby enhancing psychological resilience (Fuchs and Schlimme, 2021).

From an applied standpoint, the four core components of psychological capital—self-efficacy, hope, optimism, and resilience—can be effectively cultivated through embodied interventions: physical activity fosters self-efficacy and stress tolerance by enhancing proprioceptive feedback (Lan, 2019; Biddle and Mutrie, 2021). Artistic and creative movement promotes emotional expression and symbolic transformation, mitigating inner conflicts (Chen, 2023). Group-based bodily collaboration, such as synchronized breathing or cooperative movement, strengthens social bonding and empathy (Zhang, 2024; Gallese, 2023). Conversely, disembodiment, characterized by prolonged immobility or digital over-engagement, may generate alienation, emptiness, and loss of self-control (Fuchs, 2022).

Educational practices further validate these mechanisms. In mental-health courses adopting embodied mapping strategies, abstract psychological concepts are transformed into physically perceptible experiences (Xu et al., 2023). Likewise, contextualized classroom activities integrate cognition and emotion through bodily participation in simulated environments, promoting self-reflection and empathy (Zhuang, 2024). These approaches are consistent with the national educational vision of China Education Modernization 2035, which calls for developing students’ holistic capacities through “the integration of knowledge and action”.

In summary, embodied cognition redefines the paradigm of psychological health by establishing the body as both the foundation and the vehicle of mental adaptation. The synergy of body–mind–environment constitutes the central mechanism for mental health cultivation. Consequently, the Embodied Cognition Scale proposed in this study provides a quantitative framework to identify risks of mind–body imbalance and to guide evidence-based interventions in university settings. By localizing measurement to the Chinese sociocultural and educational context, it aligns with the broader agenda of promoting integrated physical and mental development in contemporary higher education.

## 2 Research methods

### 2.1 Instrument development process

#### 2.1.1 Qualitative exploration and theoretical anchoring

To establish the conceptual dimensions and initial item pool of the Embodied Cognition Scale (ECS), the study adopted a theory-driven and data-supported approach guided by contemporary embodied cognition frameworks (Barsalou, 2020; Shapiro, 2019; DeVellis, 2021). Following the mixed-method design, a purposive sampling strategy was applied to recruit 27 students and 8 instructors from a comprehensive university in Shandong Province. Participants were selected to represent a range of disciplinary and embodied experience backgrounds (7 arts majors, 5 physical education majors, and 15 students from general education programs). The age of student participants ranged from 17 to 23 years (*M* = 20.1, SD = 1.27), ensuring representativeness of cognitive and bodily development stages.

All interviewees provided written informed consent, and the research protocol was approved by the Ethics Committee of Heze University, in accordance with the Declaration of Helsinki. Before each interview, participants were informed of their right to withdraw and the anonymized academic use of the data. Interviews were conducted one-on-one in a semi-structured format by two trained researchers. Each session lasted approximately 20 ± 5 min, ensuring balance between data depth and participant comfort.

The interview protocol centered on the core question: “Please describe in detail a recent situation in which you regulated emotions or solved a problem through bodily activity.” Additional prompts (e.g., “What physical sensations did you notice?” “How did movement influence your thinking or emotions?”) facilitated elaboration without suggestion. All responses were audio-recorded, transcribed verbatim, and anonymized before analysis.

##### 2.1.1.1 Data processing and theoretical coding

Textual data were analyzed using thematic content analysis and word frequency visualization with MicroWordCloud 2.0. High-frequency words (*n* = 25) included “body,” “movement,” “perception,” “coordination,” “adjustment,” and “emotion.” Two independent coders categorized the terms, achieving a 0.70 inter-rater agreement rate. After multiple coding iterations, four major categories were identified:

Body perception and movement – internal awareness of physical sensations and motor fluency;

Environmental interaction and response – adaptation to contextual and spatial stimuli;

Emotional expression and transformation – outward expression and modulation of emotion through the body;

Cognitive adjustment and coordination – real-time reorganization of thought and action.

**Figure 1:**
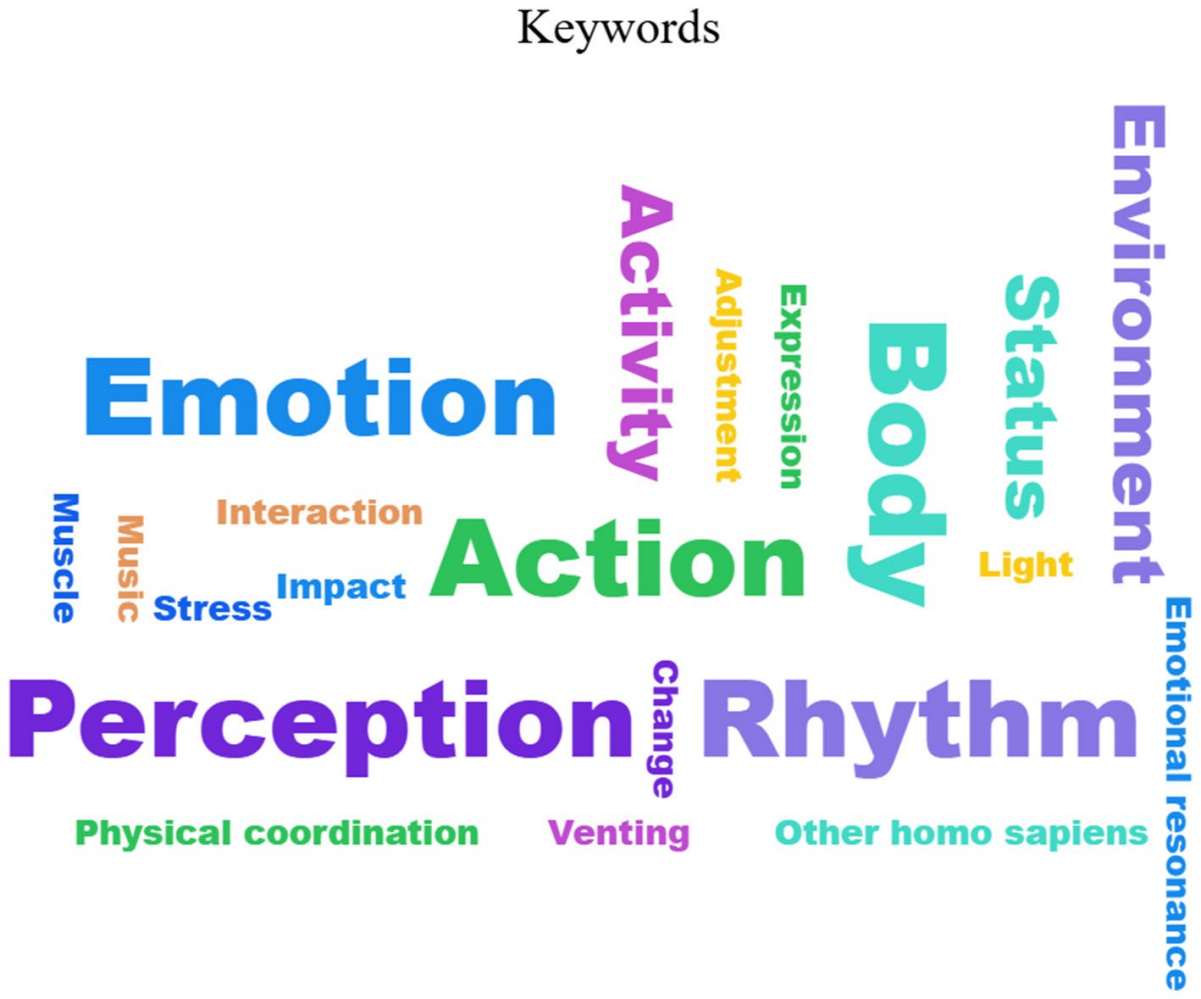
A word cloud diagram.

The coding process reached theoretical saturation after 25 transcripts, as no new conceptual categories emerged in the final two interviews. The categories were subsequently validated against the embodied cognition framework—specifically Barsalou’s (2008) “grounded cognition” and Wilson and Golonka’s (2013) six views of embodiment—to ensure theoretical coherence. Based on the dual anchoring of empirical frequency and theoretical mapping, an initial pool of 30 candidate items was formulated in Chinese, reflecting each conceptual domain. See Figure 1 for a visual representation of the most frequent embodied-cognition–related terms emerging from the qualitative interviews.

Furthermore, five experts in psychology, education, and dance studies independently reviewed the items for semantic clarity and cultural appropriateness (Content Validity Index, S-CVI = 0.92). Revisions were made following expert consensus to refine item wording and eliminate ambiguity before quantitative testing.

#### 2.1.2 Pretest

In a university in Shandong, 500 questionnaires for the initial measurement of embodied cognition were distributed to college students through convenient sampling. All the questionnaires were retrieved, with 465 valid samples, resulting in a valid response rate of 93%.

The respondents voluntarily completed the questionnaire, with an average time of about 5 min. The SPSS 27 software was used for data analysis. The principal component analysis (PCA) and the Varimax rotation method were adopted. The Listwise method was used to handle missing values, and the extraction criterion was an eigenvalue >1.

In the discriminant analysis of the items on the Embodied Cognition Scale, the scores of the analysis items are first summed up. Then, they are divided into high-score and low-score groups, with the 27th and 73rd percentiles as the boundaries. Subsequently, a T-test is used to compare the differences between the high-score and low-score groups. If there are differences, it indicates that the scale items are appropriately designed (Kelley, 1939); otherwise, it means that the scale items cannot distinguish information, and the design is unreasonable and the items should be deleted. As shown in Table 1, when analyzing items 1–30, 29 items show very obvious discrimination between the low-score and high-score groups, as all *p*-values are less than 0.01. However, for “Analysis Item 4,” *p* = 0.681 > 0.05, and the correlation with the total scale score is 0.08 > 0.02, which means that there is no good discrimination between the low-score and high-score groups for “Analysis Item 4.” The low correlation coefficient implies that “Analysis Item 4” probably needs to be deleted or modified.

**Table 1:** Results of item analysis.

	Group (mean ± standard deviation)		Correlation with the total score of the scale	*p*
	Low-score group (*n* = 125)	High-score group (*n* = 131)		
1.	3.87 ± 1.28	5.63 ± 1.42	0.465	0.000
2.	4.46 ± 1.48	6.25 ± 1.00	0.504	0.000
3.	4.56 ± 1.23	6.30 ± 1.09	0.560	0.000
4.	3.34 ± 1.42	3.44 ± 2.33	0.080	0.681
5.	3.72 ± 1.24	5.72 ± 1.28	0.563	0.000
6.	3.22 ± 1.36	4.86 ± 1.83	0.439	0.000
7.	4.11 ± 1.36	5.20 ± 1.84	0.296	0.000
8.	4.56 ± 1.33	6.18 ± 1.07	0.511	0.000
9.	4.22 ± 1.11	6.11 ± 0.99	0.635	0.000
10.	4.22 ± 1.22	5.89 ± 1.34	0.492	0.000
11.	4.31 ± 1.02	6.21 ± 0.98	0.671	0.000
12.	4.46 ± 1.01	6.40 ± 0.69	0.678	0.000
13.	4.12 ± 1.10	5.94 ± 1.23	0.583	0.000
14.	3.95 ± 1.13	5.13 ± 1.72	0.384	0.000
15.	3.92 ± 1.20	5.31 ± 1.61	0.403	0.000
16.	4.18 ± 1.12	6.15 ± 0.92	0.636	0.000
17.	3.97 ± 0.94	6.16 ± 0.92	0.702	0.000
18.	4.09 ± 0.95	6.04 ± 1.10	0.623	0.000
19.	4.14 ± 0.90	6.20 ± 0.96	0.695	0.000
20.	4.09 ± 1.11	5.97 ± 1.12	0.588	0.000
21.	3.94 ± 0.91	6.09 ± 0.96	0.722	0.000
22.	3.98 ± 0.94	6.01 ± 1.17	0.679	0.000
23.	3.96 ± 0.91	6.05 ± 1.20	0.684	0.000
24.	4.22 ± 0.76	6.36 ± 0.82	0.725	0.000
25.	4.21 ± 0.88	6.28 ± 0.81	0.732	0.000
26.	4.18 ± 0.86	6.21 ± 0.90	0.697	0.000
27.	4.09 ± 0.90	6.17 ± 0.82	0.712	0.000
28.	4.12 ± 0.89	6.28 ± 0.72	0.735	0.000
29.	4.40 ± 1.09	6.29 ± 0.81	0.647	0.000
30.	4.14 ± 0.96	6.18 ± 0.88	0.702	0.000

*p* < 0.05, *p* < 0.01.

After the discriminability analysis in the first step, factor analysis for dimensionality reduction of these 29 items was conducted in SPSS 27. The KMO test value was 0.944, indicating a significant correlation between variables. The Bartlett’s test of sphericity yielded a *χ*^2^ value of 7533.358 (*p* = 0.000). These indicators jointly verified the statistical feasibility of factor analysis, confirming that the data were suitable for factor analysis (Table 2).

**Table 2:** KMO and Bartlett’s test.

KMO measure of sampling adequacy		0.944
Bartlett’s test of sphericity	Approximate chi-square	7533.358
	Degree of freedom	406
	Significance	0.000

Five initial factors were extracted through principal component analysis, with a cumulative variance contribution rate of 61.147%, indicating that the factor structure can effectively explain the variation in the original data. The cognitive restructuring factor, which has the strongest explanatory power, encompasses items 25, 26, 27, 28, 24, 30, and 29. These items collectively reflect the function of physical activity in reshaping high-order cognitive strategies, fully aligning with the “cognitive regulation” dimension in the theoretical framework. The environmental interaction factor consists of items 14, 15, 7, and 13, highlighting the dynamic interaction between environmental stimuli and bodily perception, which echoes the integration theory of “body and environment.” The emotional concretization factor includes items 18, 19, 22, 21, 20, and 8, systematically representing the concrete path of emotional expression through the body, in line with the theoretical concept of “emotional expression.” The social synchronization factor integrates items 9, 11, 10, and 12, revealing the coordination mechanism of the body in social situations, corresponding to the theoretical dimension of “social embodiment.” The fundamental bodily perception factor is composed of items 2, 3, 1, and 17. These physiological perception items form the underlying basis of embodied cognition, consistent with the theoretical assumption of “body awareness.”

During the model optimization process, items 5, 6, and 16 were removed due to validity defects where their loading values were lower than 0.4 or there were cross-factor loadings > 0.3. Item 23 exhibited cross-factor characteristics in the cognitive and emotional dimensions, empirically supporting the “dual-channel” theory of embodied cognition. Item 17 was incorporated into the body perception factor, strengthening the connection between physiological mechanisms and cognitive functions. The final five-factor model with 26 items not only meets the statistical requirements but also perfectly aligns with the theory of embodied cognition, integrating multiple aspects such as its physiological basis, environmental interaction, emotional expression, cognitive regulation, and social coordination (Table 3).

**Table 3:** Rotated component matrix A.

	1	2	3	4	5
1.					0.669
2.					0.763
3.					0.666
5.					
6.					
7.				0.659	
8.			0.683		
9.			0.647		
10.			0.585		
11.			0.594		
12.			0.573		
13.				0.614	
14.				0.846	
15.				0.807	
16.					
17.		0.596			
18.		0.651			
19.		0.629			
20.		0.564			
21.		0.596			
22.		0.643			
23.	0.518	0.549			
24.	0.737				
25.	0.789				
26.	0.809				
27.	0.807				
28.	0.775				
29.	0.695				
30.	0.736				

Factor name	Cognitive restructuring	Emotional visualization	Action mirroring	Environmental interaction	Physical perception
Characteristic root	5.692	3.327	2.711	3.656	2.346
Explained variance	19.627	11.471	9.349	12.608	8.091
Cumulative explained variance	19.627	43.707	53.067	32.236	61.147

Through the confirmatory factor analysis of 465 samples using SPSSAU, the study tested 5 factors related to embodied cognition. First, the sample size was sufficient, the discriminant validity was good, and the overall model fit basically met the standard. However, there were deficiencies in the convergent validity. The AVE values of F1, F2, and F3 were all lower than 0.5, and the standardized loading coefficients of 5 measurement items were lower than 0.6. Meanwhile, there was a high correlation among F3, F4, and F5 (Table 4). In summary, the model structure is basically valid but needs optimization. It is recommended to remove the low-loading items and recheck the theoretical rationality of factor division.

**Table 4:** Results of the AVE and CR indicators of the model.

Factor	Average variance extracted (AVE) value	Composite reliability (CR) value
F1	0.458	0.713
F2	0.475	0.780
F3	0.455	0.803
F4	0.509	0.878
F5	0.682	0.937

By deleting five low-load items and renaming the factors based on the embodied cognition theory, the names of the refactored factors systematically reflect the integrity of the unity of the mind and body. “Physical physiological perception” directly corresponds to the fundamental role of the body in cognition, emphasizing the shaping of cognition by physiological signals (Damasio, 1999). It highlights the physiological mechanism more than the original “body perception.” “Social embodiment” replaces “environmental interaction,” encompassing social embedding in the environment and reflecting the adaptive adjustment of the body in social situations (Gallagher, 2013). “Embodied perception and imitation” integrates “action mirroring,” emphasizing that observational learning is achieved through the simulation of the body’s motor system (Gallese and Goldman, 1998), revealing the action essence of cognition. “Emotional embodiment” deepens “emotional concretization,” indicating that emotions are specifically experienced through somatic markers (Niedenthal, 2007), highlighting the inseparability of emotions and body states. “Cognitive reconstruction” is retained because its essence is still the reinterpretation of meaning based on embodied experiences (Lakoff and Johnson, 1999). However, within this framework, its operating mechanism clearly depends on the embodied basis. The overall refactoring enables the names of each factor to logically and consistently map the multi-dimensional interactions of embodied cognition: physiological basis, environmental embedding, motor system, emotional experience, and higher-order integration (Shapiro, 2019), resulting in an embodied cognition scale with five factors and 21 items. The scale meets the requirements of both convergent validity and discriminant validity. Although there are phenomena of locally high residuals and potential conceptual overlap between “emotional embodiment” and “cognitive reconstruction,” the model fit indices reach an acceptable level (Table 5).

**Table 5:** Results of the AVE and CR indicators of the model.

Factor	Average variance extracted (AVE) value	Composite reliability (CR) value
F1	0.552	0.709
F2	0.518	0.762
F3	0.551	0.786
F4	0.530	0.871
F5	0.681	0.937

To validate the rationality of the five-factor structure of the scale, all 21 items were regarded as a single factor, and confirmatory factor analysis was used to conduct goodness-of-fit tests on the single-factor model and the five-factor model, respectively. The results showed that the fit indices of the single-factor model indicated poor goodness of fit, suggesting that this model failed to adequately fit the data and reflecting the irrationality of the single-factor structure. In contrast, the fit indices of the five-factor model were significantly improved and reached or approached the ideal standards, confirming that the five-factor structure had stronger theoretical rationality and statistical goodness of fit (Table 6). Therefore, the five-factor model outperformed the single-factor model and provided robust support for the construct validity of the scale.

**Table 6:** Fitting indicators of factor analysis.

	*χ*^2^/df	GFI	IFI	TLI	CFI	RMSEA	AVE	HTMT
Single-factor model	8.027	0.697	0.774	0.748	0.773	0.123	0.422	
Five-factor model	3.443	0.886	0.926	0.912	0.925	0.013	0.518–0.681	0.268 ~ 0.794
Goodness-of-fit indices	<4	>0.9	>0.9	>0.9	>0.9	<0.08	>0.5	<0.85

The optimized model meets the psychometric requirements, supporting large-sample confirmatory analysis to test cross-sample stability. Further exploration of the interaction paths between factors is needed through structural equation modeling.

#### 2.1.3 Validation with large samples

**Figure 2:**
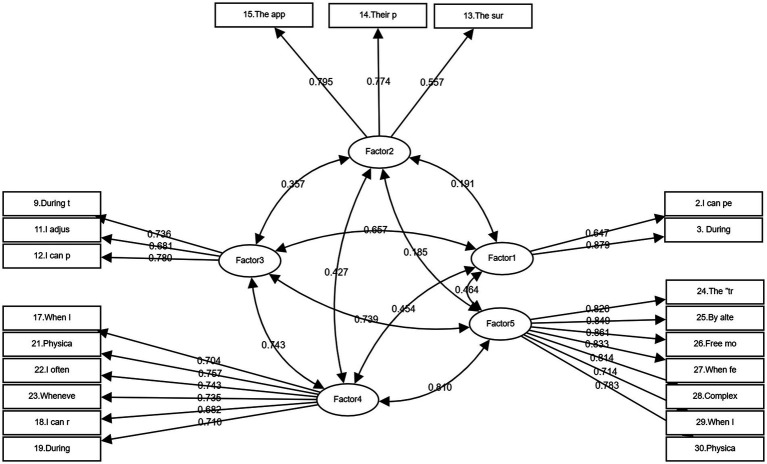
The structural model and standardized path coefficients.

A large-sample data of 1,000 questionnaires were distributed to universities in multiple regions across the country through random sampling, and 918 were recovered, with an effective rate of 91.8%. Confirmatory factor analysis was conducted using Amos software. In terms of convergent validity, the average variance extracted (AVE) values of all latent variables were higher than 0.50, and the composite reliability (CR) values were greater than 0.70, confirming that the observed variables could effectively represent the corresponding latent variables and the internal consistency of the scale met the standard. The HTMT heterotrait – monotrait ratio values were all lower than the conservative threshold of 0.85, further supporting the sufficient discriminability between latent variables. The key indicators of model goodness-of-fit all reached the academic standards (*χ*^2^/df = 4.86 < 5, CFI = 0.928 > 0.90, RMSEA = 0.062 < 0.08, SRMR = 0.068 < 0.08). Although the AGFI was slightly lower than 0.90, the TLI and IFI corroborated that the overall fit of the model was acceptable. Although the standardized loading (0.557) of item 13 is slightly low, its SMC value still exceeds the critical criterion of 0.25 (Hair et al., 2019). Moreover, Chinese college students may exhibit cultural specificity in their sensitivity to the environment and the body. Deleting this item would undermine the ecological validity and cultural representativeness of the scale. The item’s expression has been optimized through a clear operational definition. The structural model and standardized path coefficients are presented in Figure 2. In summary, both the validity and reliability of the revised model meet the psychometric standards, making it suitable for cross-regional research on body perception among Chinese college students. Additionally, the control of gender ratio enhances the representativeness of the sample.

### 2.2 Criterion-validity test

In order to test the external validity of the scale, Chen and Jiang’s (2007) Body Consciousness Scale for College Students (BCS) was used as the criterion for empirical analysis. Pearson correlation analysis showed that there was a strong correlation between the personal cognition scale and BCS total score (*r* = 0.81, *p* < 0.01). Body perception correlated 0.72 with BCS internal body consciousness dimension, and emotion regulation correlated 0.75 with BCS emotional body association dimension (*p*s < 0.01). This result confirms that the scale can effectively capture the theoretical covariant characteristics of embodied cognition and body consciousness, and the correlation of key dimensions is particularly prominent, which provides strong support for the empirical validity of the scale.

### 2.3 Reliability analysis

#### 2.3.1 Internal consistency analysis

SPSSAU was used to analyze 918 valid samples of formal version of embodied cognition scale. Physical physiological perception 0.742, social embodiment 0.756, embodied perception and imitation 0.777, emotional embodiment 0.867, cognitive reconstruction 0.931, CR value of total scale 0.954, all indexes were higher than psychometric standard 0.70, indicating excellent internal consistency reliability of scale.

#### 2.3.2 Test–retest reliability analysis

In the test – retest reliability analysis, a test with a two-week interval was adopted. The Embodied Cognition Scale was administered to a sample of 158 college students. The Pearson correlation coefficient and the intra-class correlation coefficient (ICC) were calculated to evaluate the temporal stability. The results showed that the test – retest correlation coefficient of the total scale reached 0.94 (*p* < 0.01). Each dimension, such as body perception (*r* = 0.86) and emotional regulation (*r* = 0.89), demonstrated a high degree of consistency. The ICC values ranged from 0.76 to 0.93, far exceeding the psychometric standards. In addition, the measurement invariance test confirmed that the factor structure was equivalent across time. The residual variance analysis showed no significant time effect. Ultimately, it indicated that the scale has excellent cross-time reliability and stability and is suitable for long-term follow-up studies.

## 3 Fit between the scale structure and the theory

The final five-dimensional structure of the Embodied Cognition Scale (ECS) aligns closely with the contemporary theoretical framework of embodied, enactive, extended, and situated cognition (Varela et al., 1991; Shapiro, 2019). Each dimension represents a distinct but interrelated aspect of how cognition is grounded in bodily, affective, and environmental interactions, reflecting both universal embodied mechanisms and the contextual characteristics of Chinese university students.

The dimension of bodily perception operationalizes the premise that the body provides the material substrate of cognitive representation (Barsalou, 2020; Damasio, 1999). According to grounded cognition theory, sensorimotor signals and interoceptive feedback form the basis of conceptual understanding. Empirical studies show that proprioceptive sensitivity and kinesthetic awareness significantly predict attentional stability and emotional regulation (Craig, 2009; Mehling et al., 2022).

In the ECS, this dimension is measured through items that assess awareness of physical sensations (e.g., muscle tension, respiration, and somatic rhythm), movement coordination, and body–environment attunement. These items capture the fundamental mechanism whereby bodily states constrain and enable cognitive processing. The findings support Ye’s (2011) argument that the structure and activity of the body delimit the scope of cognition and align with Ding’s (2023) psychometric evidence that reduced somatic awareness leads to cognitive rigidity in sedentary students. Thus, this factor concretizes the theoretical proposition that “physiological experiences shape cognitive representations.”

The social embodiment factor corresponds to the extended and situated cognition perspectives (Clark and Chalmers, 1998; Gallagher, 2013), which posit that cognition extends beyond the individual mind into social and environmental systems. Bodily gestures, interpersonal synchrony, and spatial coordination form the substrate for empathic understanding and social reasoning (Gallese, 2023). Neuroimaging research demonstrates that interpersonal motor synchronization activates shared neural circuits within the mirror neuron system, facilitating mutual prediction and trust (Redcay and Schilbach, 2023).

In this study, items related to spatial adaptation, interpersonal distance regulation, and joint body movement operationalize social embodiment. These behaviors represent how individuals dynamically negotiate meaning through embodied communication—a concept consistent with Confucian notions of “礼以体达” (bodily propriety in social harmony). Empirically, Wang et al. (2022) verified that synchronized movement enhances collective creativity by promoting inter-brain coherence in the prefrontal cortex. Therefore, the social embodiment dimension embodies the principle that “the mind is socially extended through bodily co-regulation.”

The embodied imitation dimension reflects the enactive approach, emphasizing that cognition arises from sensorimotor enactment and bodily simulation (Varela et al., 1991; Di Paolo et al., 2017). Mirror neuron research provides the neurobiological basis for this process, indicating that observing or imagining an action activates neural substrates similar to those used in performing the action itself (Gallese and Goldman, 1998; Huang et al., 2021).

Within educational and artistic contexts, imitation and mirroring serve as mechanisms for embodied learning and creative transformation (Li and Xiong, 2021; Pfeiffer et al., 2021). The ECS captures this process through items assessing rhythm synchronization, motor resonance, and intention inference. These indicators represent how learners internalize knowledge through embodied simulation, transforming perception into action-based understanding. This dimension thus operationalizes the enactive claim that “cognition is enacted through movement and imitation.”

The dimension of emotional embodiment corresponds to the emotion–body bidirectional mapping theory, which posits that emotional understanding involves bodily simulation and feedback (Niedenthal, 2007; Strack et al., 1988). Facial expressions, posture, and movement patterns not only reflect but also regulate affective states (Peper et al., 2021).

In this scale, items capture individuals’ awareness of emotional cues expressed through movement, bodily responses to mood changes, and capacity for expressive regulation. These indicators quantify the embodied affective mechanisms underlying psychological resilience (Fuchs and Schlimme, 2021). For example, in dance movement therapy, emotional transformation occurs through kinesthetic empathy and sensorimotor resonance (Bräuninger, 2014; Cheng, 2022). Ye and Su (2024) further emphasize that embodied emotional training can restore equilibrium in the “body–mind–environment” system of college students. Consequently, this dimension embodies the theoretical assertion that “emotions are not merely felt but physically enacted and perceived.”

The cognitive reconstruction dimension integrates the higher-order cognitive outcomes of embodied activity. From an embodied cognition perspective, action execution modulates executive function and cognitive flexibility by reducing prefrontal over-control and activating associative networks (Wang et al., 2019; Glenberg, 2010). Theoretically, this reflects the body schema mechanism—where physical experience reorganizes mental representation (Gallagher, 2005; Shapiro, 2019).

In this study, this factor includes items measuring metaphorical thinking through action, reframing of failure experiences, and adaptive learning through movement. These reflect the educational significance of embodied cognition: movement becomes a medium for conceptual change, creative thought, and meaning reconstruction. Zhang (2024) demonstrated that physical disorder in one’s environment activates creative reorganization of cognitive patterns through metaphorical embodiment. Therefore, this factor operationalizes the principle that “physical actions reshape abstract cognition through generative learning.”

Together, these five factors reflect a hierarchical structure of embodied cognition—from physiological grounding to social extension, affective resonance, and cognitive integration. The structural model verified through CFA and SEM empirically supports the theoretical continuum proposed by Barsalou (2020) and Shapiro (2019).

Moreover, the ECS introduces a cultural–psychometric innovation by embedding the Confucian ideals of “unity of body and mind” and “integration of knowledge and action” into its framework. This localization bridges Western cognitive science and Chinese holistic philosophy, providing a culturally congruent tool for evaluating embodied learning and mental health in the university context.

Finally, by aligning empirical factor evidence with multi-level theoretical constructs, the ECS achieves a balanced integration of conceptual validity and psychometric rigor. It offers not only a diagnostic framework for embodied cognition but also a foundation for intervention programs aimed at cultivating students’ holistic well-being and adaptive intelligence.

## 4 Discussion and outlook

### 4.1 Theoretical contributions

The present study contributes to the field of embodied cognition and psychological assessment in several important ways.

First, it provides a localized theoretical operationalization of embodied cognition by empirically confirming a five-dimensional structure—bodily perception, social embodiment, embodied imitation, emotional embodiment, and cognitive reconstruction. This configuration reflects the integrative continuum of cognitive grounding: from the sensorimotor basis to affective regulation and adaptive cognition. The finding aligns with Barsalou’s (2020) grounded cognition model and extends it by incorporating cultural–contextual embodiment rooted in Confucian and aesthetic education traditions.

Second, the ECS represents a methodological innovation in operationalizing an abstract construct into a psychometrically robust instrument. Through a multi-phase validation process—qualitative conceptualization, exploratory refinement, and confirmatory modeling—this research demonstrates that embodied cognition can be quantitatively measured without reducing its ecological and experiential richness. It bridges the gap between philosophical abstraction and empirical measurement, addressing one of the longstanding criticisms of the EC paradigm (Mahon and Caramazza, 2008).

Third, the ECS enriches cross-disciplinary dialog by integrating insights from psychology, education, and movement science. The inclusion of both emotional and cognitive embodiment dimensions responds to the current trend of embodied affective science (Fuchs, 2022), providing a conceptual framework to explore the links among body awareness, emotional regulation, and resilience.

In doing so, this study reaffirms the principle that the body is not merely an object of cognition but a constitutive medium of thought, emotion, and adaptation.

### 4.2 Practical implications

The validated ECS has broad applicability across education, mental health, and organizational contexts. Within higher education, the scale can be used as a diagnostic tool to assess students’ embodied learning readiness and bodily engagement in classroom contexts. For example, low scores in bodily perception may indicate limited somatic awareness and risk of academic fatigue, while deficits in social embodiment could reflect reduced empathy and interpersonal attunement. By integrating ECS-based diagnostics into curriculum design, educators can introduce embodied teaching methods such as active learning, movement-based reflection, and art-integrated instruction. These interventions are aligned with the strategic objectives of China Education Modernization 2035, emphasizing holistic mind–body education and student-centered learning reforms.

In psychological counseling and university mental-health programs, the ECS can function as a screening and monitoring instrument to detect mind–body imbalance, low emotional embodiment, or cognitive rigidity. It enables practitioners to design embodied intervention protocols, such as mindfulness body scanning, expressive movement therapy, and group-based somatic awareness training. These embodied approaches have been empirically shown to enhance resilience, reduce stress, and improve affective flexibility (Kabat-Zinn, 2003; Bräuninger, 2014; Cheng, 2022).

Moreover, the ECS can complement existing scales like the Connor–Davidson Resilience Scale (CD-RISC) or the Body Consciousness Scale by offering a multidimensional diagnostic profile that connects bodily and psychological indices.

At the macro level, this research provides quantitative evidence for educational and health policy planning. In the context of rising academic stress and sedentary lifestyles among Chinese college students, the ECS offers measurable indicators for evaluating the effectiveness of campus physical–psychological integration programs. It can serve as a foundation for designing institutional initiatives that integrate embodied learning, physical education, and psychological resilience training, thereby promoting “Healthy China 2030” and “Education Modernization 2035” goals.

Furthermore, the cultural adaptability of the ECS opens avenues for cross-cultural comparison: the scale may be translated and adapted for international use to explore how embodied cognition manifests in different cultural systems of body–mind conceptualization.

### 4.3 Limitations and future directions

Despite its theoretical and practical significance, this study has certain limitations that point to directions for further research.

First, the study employed a cross-sectional design, which limits the ability to infer causal relationships between embodied cognition and psychological outcomes such as resilience or well-being. Future research should employ longitudinal and experimental designs to explore developmental changes in embodied cognition and its predictive effects on adaptation and learning.

Second, although the sample included students from diverse regions of China, further cross-cultural validation is needed to test measurement invariance across different linguistic and cultural contexts. This would enable the ECS to become a standardized international instrument for studying embodiment.

Third, while the current model captures the five core dimensions of embodied cognition, neural and physiological data were not integrated into the measurement framework. Future studies could combine self-report, behavioral, and neurophysiological indices (e.g., heart-rate variability, EEG, fNIRS) to construct a multi-modal assessment system.

Finally, practical applications of the ECS should be further examined in intervention studies, such as embedding embodied cognition training into university curricula, or implementing embodied learning workshops in counseling centers. Through such interventions, researchers could examine how improvements in specific ECS dimensions translate into gains in resilience, emotional regulation, and academic engagement.

### 4.4 General outlook

This research marks a significant step toward quantifying the unquantifiable—transforming the abstract philosophical concept of embodied cognition into a psychometrically sound and culturally meaningful scale. It reaffirms that cognition, emotion, and social interaction are embodied processes that constitute the foundation of human learning and adaptation.

Looking ahead, the ECS may serve as a cornerstone for the integration of embodied cognition into national mental-health education systems, supporting the shift from “knowledge transmission” to “whole-person cultivation.” By linking the scientific study of embodiment with educational innovation, clinical practice, and policy development, the ECS contributes to a new vision of education that recognizes the body as a site of cognition, culture, and care.

## Data Availability

The original contributions presented in the study are included in the article/supplementary material, further inquiries can be directed to the corresponding author.
